# Molecular Tactics of Biocontrol Fungi to Hack Plant Immunity for Successful Host Colonization—A Focus on *Trichoderma* Fungi

**DOI:** 10.3390/microorganisms13061251

**Published:** 2025-05-28

**Authors:** Yingfen Yang, Meiwei Zhao, Guotao Li, Ying Wang, Qingqing Shen, Jun Yang, Tahani A. Y. Asseri, Yanjun Wang, Min Guo, Waqar Ahmed

**Affiliations:** 1 Academy of Science and Technology, Chuxiong Normal University, Chuxiong 675000, China; 2 Yunnan Urban Agricultural Engineering and Technological Research Center, College of Agronomy, Kunming University, Kunming 650214, China; 3 College of Resources, Environment, and Chemistry, Chuxiong Normal University, Chuxiong 675000, China; 4 Department of Biology, College of Science, King Khalid University, Abha 61413, Saudi Arabia; 5 Personnel Office, Chuxiong Normal University, Chuxiong 675000, China; 6 Guangdong Province Key Laboratory of Microbial Signals and Disease Control, College of Plant Protection, South China Agricultural University, Guangzhou 510642, China

**Keywords:** signaling, defense, RNAs, metabolomics, genomics

## Abstract

To play a role effectively, biocontrol fungi must fight against plant immune response and establish a symbiotic interaction with their host. After successfully colonizing the host plant, the biocontrol fungi may deliver beneficial effects related to plant health and resistance against phytopathogens. These fungi use a variety of tactics to bypass the host immune response, including the production of effector proteins, miRNA interference, manipulation of host defense mechanisms, and others. In this review article, we discussed these strategies of biocontrol fungi based on recent findings. These methods enable the fungi to escape the plant’s intrinsic immunity and finely adjust the plant’s defense signaling cascades. Additionally, we discussed the importance of the physical barrier in the form of host cell walls and elucidated how biocontrol fungi use a combination of mechanical and enzymatic tactics to overcome this obstacle. Given the evolving comprehensions from molecular biology, genomics, and ecology, this review article highlights the prospective for a holistic, interdisciplinary approach to improve our understanding of the biocontrol mechanism.

## 1. Introduction

The intricate communities of microflora associated with plants may be harmful and beneficial for the host plants. Among these microbes, fungi may prove to be hazardous, causing different diseases; on the other hand, many of these not only improve plant health and growth but also increase the procurement of nutrients and plant tolerance against several abiotic and biotic stresses using a variety of different mechanism [1]. These fungi help the plants manipulate the harmful impacts of biotic factors without damaging the host plant and are designated as biocontrol fungal endophytes. They colonize numerous plant species growing in maneuvered or natural ecosystems. These fungal biocontrol agents are believed to be vital partners of colonized host plants, playing a pivotal role in enhancing tolerance against stress compared to hosts lacking these symbionts [2]. Most plant-colonizing fungi do not have any specified impact; nonetheless, various fungi substantiate either symbiotic or pathogenic affiliation with the host plants. Primarily, the consequences of the relationship are highly dependent on prevalent environmental conditions, the type of the colonizing fungal agent, and the host colonized [3]. In addition, Plant-fungus interactions, whether symbiotic or pathogenic, are also influenced by the microbiota present in the rhizosphere or phyllosphere [4,5,6,7,8,9,10,11].

The host plants can recognize the microorganisms through pathogen/microbe-associated molecular patterns (PAMPs/MAMPs) via pattern recognition receptors (PRRs). These receptors are a group of recognition proteins present on the cell’s surface and are employed in the primary signals that induce the initial line of the plant’s intrinsic immunity system [12]. Generally, while establishing the relationship majority of the pathways of host defense systems triggered by miRNAs are inactivated; otherwise, that would have hampered the establishment of fungal biocontrol agents as well [13]. These microbial biocontrols are associated with numerous plant species irrespective of their origin. The potential of these biocontrols to enter, colonize, and proliferate in the plants makes this novel, exhibiting multifaceted relationships within the plants. Several host plant actions are affected by these fungal biocontrols. The biocontrol microbes enhance plant growth, act as ameliorators of abiotic stresses, and evoke the plant defense system against biotic agents [14]. Nonetheless, to materialize these beneficent roles, the fungal biocontrol agents must overcome the vital challenges of escaping the complex intrinsic defense system and colonizing the root system. They need to overcome physical barriers (cuticle, cell wall), compete with other microbes in the rhizosphere and plant tissues for space and nutrients, and be adaptable to the environment and secondary antifungal metabolites in the host. The current review aims to determine the exquisite pathway and the sophisticated approach fungi use to respond to and regulate the host defense reactions to establish symbiotic relationships.

The first part of the review article will emphasize the basic perception of the plant’s intrinsic defense system, investigating the numerous defense pathways involved and its multifaceted structural barriers. Based on this context, we will explore the biochemical sensing strategies that assist the two-way interaction between the host and the fungal biocontrol agents. A significant part of the review will be dedicated to molecular mimicry, a clever approach used by the fungal biocontrol agent to intervene with the host defense detection systems. The later part will focus on special secretion pathways, encompassing the secretion of apoplastic proteins and the nucleotides used by the fungal biocontrol to alter the host’s internal environment and improve host tissue colonization. Moreover, efforts will be made to focus on the transcriptional modifications that transform the plant’s hormonal defense system, efficiently changing the host biochemical milieu supporting fungi colonization. The review will also include a detailed investigation of how fungal biocontrol agents release usual symbiotic signaling factors vital in triggering and maintaining the symbiosis. In addition, the discussion will be made on the approaches used by fungal biocontrol to address the architectural intricacies of the host cell wall. The review will also discuss how the genetics of biocontrol fungi and host plants contribute to root colonization efficiency, highlighting the involvement of genes in gene theory.

The main purpose of this review article is to provide a comprehensive understanding of multilayered approaches used by fungal biocontrol to subdue the host plant defense for efficient colonization. These perceptions are vital for practical employment, advancements in the scientific field, and knowledge of sustainable environment and agriculture science. Employing an interdisciplinary approach to integrate genomic, ecology, and molecular biology, we fulfilled the shortcomings of our comprehension of the subject and encouraged future contributions in this fast-growing field of science.

## 2. Plant Immune Response; An Overview

**Figure 1:**
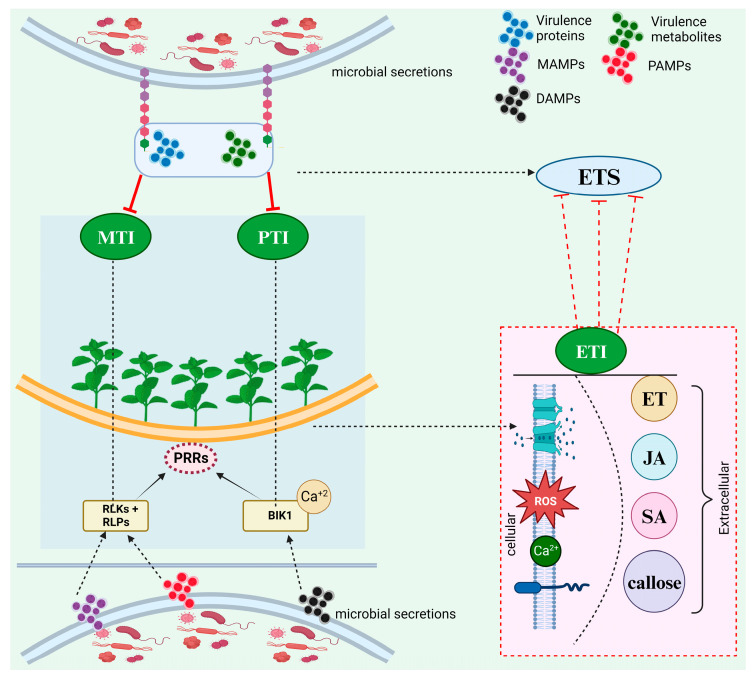
Activation of plant immunity. Generally, the MAMPs, PAMPs, and DAMPs are recognized by RLKs and RLPs as PRRs. This immune response activated by PRRs is termed MTI or PTI. In response, the pathogens also secrete various effectors (virulence proteins and metabolites) to suppress MTI and PTI and initiate promising invasion, termed ETS. The host plants trigger another defense system, ETI, which includes enhanced apoplast ROS and cytosolic Ca^2+^ levels, producing plant hormones like SA, JA, and ET, callose deposition.

Both pathogenic and beneficial microbes intrude into the host without damaging the plasma membrane to procure nutrients. Genetically, plants have an intrinsic defense system that reacts to the PAMPs or MAMPs and effectors of the pathogens [15]. Phyto-pathogens release a variety of metabolites and virulence proteins known as effectors that evolved to support infection by pathogens. However, a fraction arbitrarily triggers receptors responsible for activating defense response [15]. PAMPs and MAMPs, as well as DAMPs recognition extracellularly steers to the initial level of inductive protection coined as PTI (pattern triggered immunity) that is pivotal in restricting the unaccustomed microorganisms from colonizing the host plants and in preventing invasion of pathogenic organisms onto nontolerant host plants [16]. Recognition of host- and microbe-derived patterns, as the initial level of plant defenses, is done through the deployment of a substantial amount of RLKs (receptor-like kinases) and RLPs (receptor-like proteins) as PRRs (Figure 1). Later on, the immune response initiated by PRRs is termed MTI (MAMP-triggered immunity). PTI can also be triggered through internal signals initiated by the cells’ disintegration, which is called DAMPs. PRRs also detect DAMPs and thus have a vital role in the intrinsic defense of internal elicitors or non-specialized microbes [16]. Compounds like cyclodextrin and oligogalacturonides resulting from cell wall degradation and material from damaged, necrotic, or stressed cells such as small peptides and cutin monomers may also behave as DAMPs signals in plants. Microbial peptides are also useful as inducers of plant defense [17]. In addition, as a result of the invasion, BIK1 (botrytis-induced kinase1), an effector kinase of the PRRs, can be triggered, inducing an enhancement of CNGC-mediated cytosolic calcium (Ca^2+^) that acts as a vital signal to induce PAMPs-triggered defense in plants amidst PTI [18].

The pathogens also recruit many effectors to subdue PTI and initiate promising invasion, coined ETS (effector-triggered susceptibility). In such cases, the host plants trigger another defense system, exhibiting a robust and developed type of immunity program primitively inside the host plant cell, which is named ETI (Effector-triggered immunity). Hence, ETI and PTI can trigger immunity against microbes invading the plants through a change in the ion flux all over the cell membrane, enhancing the apoplast ROS and cytosolic Ca^2+^ levels, the proliferation of NO (Nitric oxide) and triggering MAPK (Mitogen-activated protein kinase, with subsequent production of plant hormones like salicylic acid (SA), jasmonic acid (JA), and ethylene (ET), callose deposition, stomata closure, metabolic reprogramming and defense-associated transcription [19]. Other additional immune reactions activated by PTI and ETI include defense mechanisms at cellular levels (cell wall thickening, lignin accumulation), histological (abscission tissues), biochemical (hypersensitivity reaction), and genetic (activation of antifungal protein-coding genes). These mechanisms allow the plant to expand and modulate its defense against microorganisms and infection scenarios [20,21,22,23]. Hence, the first line of defense action deduced from host plant cells can differentiate between endophytes and pathogens in screening modulated by MAMP. In addition, pattern-induced defense signaling in plants, like CAD1 and MIN7, present in most of the terrestrial plant pedigree, is more likely a vital factor of genetic lineage amongst which land plants manage the level and promote the endophytic microbe diversity for health and survival in a microbe prolific environment. Besides the given mechanisms, the liquid compartment of plants termed extracellular vesicles (EVs) can transport signaling lipids and proteins, metabolites, and RNA between cells, behaving as predominant modulators of host plant-microbe interactions and exhibiting antagonism during stress. Amidst plant immunity response, the cells release EVs where micro RNAs and siRNAs from the host plant are capable of silencing the stress response and fungi genes, depicting that trans-kingdom RNA interference can also be modulated by EVs [24].

Furthermore, host plants can release phytohormones and root exudates comprising secondary metabolites such as flavonoids, alkaloids, amino acids, triterpenes, and organic acids that may act as signaling factors, stimulants, and attractants, as well as repellents or inhibitors controlling potential biotic infection and minimize the herbivore potential [25]. Structural barriers are another line of the host plant defense system, including HR (Hypersensitivity response, also called programmed cell death), cell wall thickening, and callose formation [26]. Infection from the pathogen and its establishment on susceptible hosts is prevented because of these structural barriers. A few plants may have hydrolytic enzymes such as myrosinase, which are known to break sulfur and glucose bonds in glucosinolates and generate isothiocyanates, which are toxic to pests and pathogens. Cruciferous plant leaves have large quantities of these enzymes. Besides these enzymes, plants may also have amassed other chemicals, including phenylpropanoids, flavonoids, and terpenoids [27]. Jasmonic acids, salicylic acids, and ethylene are also among other chemicals that induce resistance against the attack of plant pathogens [28]. Additional compounds that induce resistance in plants include abscisic acid (modulates defense against certain pathogens), oxalic acid, flavonoids, and phenylpropanoids [29,30,31].

## 3. Biochemical Sensing Between Biocontrol Fungi and Host Plant

Communicating between the host plant and fungal biocontrol is vital to formulate a symbiotic relationship. Nonetheless, except for a few physical properties, most of the signals used for communication are chemical factors such as peptides, volatile metabolites, lipids, polysaccharides, and peroxidase enzymes. During plant-microbe interaction, the biocontrol fungal recognition receptors like apoplastic proteins, components of the cell wall, and eATP can trigger symbiotic signals [32,33]. As biocontrol agents, Fungi must establish symbiotic interaction with host plants through successful colonization. It starts with locating the host plant roots, which is vital for successfully colonizing the roots. This subsequently results in biochemical recognition and retaliating to stimulus between the biocontrol fungi and host plant, ultimately leading to fungi penetration inside the root system for successful colonization. This will accomplish the symbiotic relationship involving the management of pests and diseases in plants [34].

**Figure 2:**
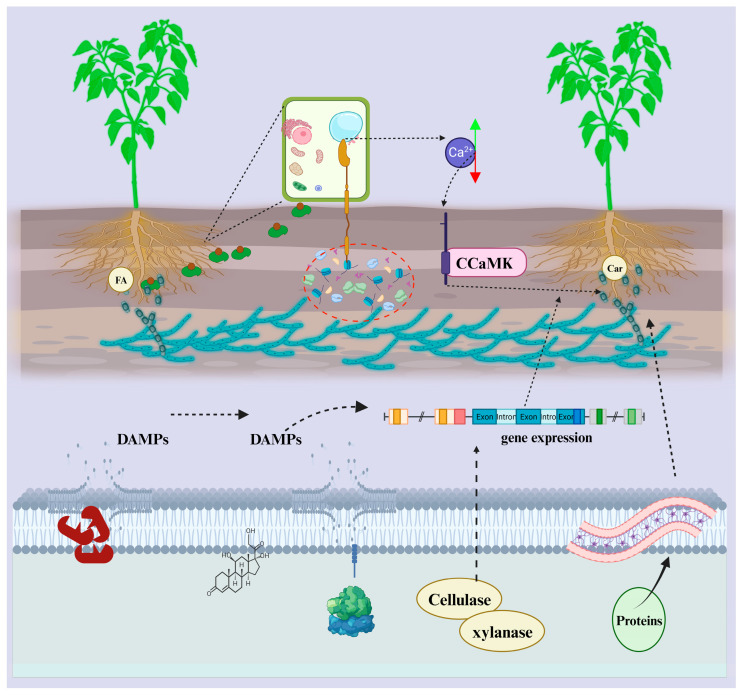
Biochemical sensing between biocontrol fungi and host plant for effective root colonization. The phytochemical signals, strigolactones, 2-hydroxyl fatty acids, and flavonoids are released by plant cells. In response to the host signals, the fungal biocontrol agents move toward the signal source and release microbe factors recognized by host receptors, resulting in signaling from the cytoplasm to the nucleus. This signaling causes the root cells of the host plant to release cytosolic calcium, the concentration of which alternates in the nucleus and cytoplasm repeatedly. The fluctuation of calcium concentration results in a concentration gradient that is decoded through protein Kinase (CCaMK), the activity of which results in improved colonization of roots by the fungal biocontrol. The biocontrol fungi secrete cell wall degrading enzymes, and CAZymes assist in the release of DAMP signal compounds. Biocontrol fungi release xylanases, and cellulases activate genes related to polysaccharide metabolism, improving root colonization. Proteins secreted by biocontrol fungi could also help enhance the cell wall’s flexibility; incomplete digestion of the middle lamella’s pectic layer would allow fungi growth between the cells, thereby increasing fungal colonization.

At the pre-colonization stage, the host plant releases phytochemical signals, termed strigolactones (Figure 2). It is a carotenoid-based plant hormone that enhances metabolism, development, and hyphae branching in fungi. It was discovered for the first time in petunia hybrid and is believed to be transported out of the plant by a demarcated exporter PDR1 (pleiotropic drug resistance 1) via suberin-free HPCs (Hypodermal passage cells) located in the plant root exodermis-layer. PDR1 is known to play a role in building up the strigolactones in plant rhizosphere zones [35]. Strigolactones are released endogenously in plant roots in a few plants devoid of apoplastic hydrophobic diffusion hurdles, like *Medicago truncatula*. They are diffused in the rhizosphere without the help of a transport system or unique exporters [36]. Flavonoids and 2-hydroxyl fatty acids are chemically secreted molecules that improve the hyphal tip’s growth, branching, and elongation [37]. Retaliating to the host signal factors, the fungal biocontrol agents grow towards the signal source and release a complex of chemicals collectively termed microbe factors.

In addition, in the symbiotic host-fungus relationship, cytokinins and oxilipines are involved in communication [38,39]. Host plant receptors recognize these factors through phosphorylation and transmit signals from the cytoplasm to the host nucleus. As a result of the detection of chemical signals sent by microbes through receptors of microbial factors, root cells of the host plant release cytosolic calcium, the concentration of which alternates in the nucleus and cytoplasm repeatedly. The oscillation of calcium concentration results in a concentration gradient that is decoded through protein Kinase (CCaMK), the activity of which, along with transcription factor (CYCLOPS) phosphorylation, results in improved colonization of roots by the fungal biocontrol [40,41]. Immunological tools help the plants ward off, control, and destroy the infection caused by incoming microorganisms that may pursue plant colonization. The intrinsic defense-signaling network is triggered upon contact with the exotic chemical molecules that may cause host cell wall degradation or when it comes with microbial contact. Plants respond to the introduction of effectors by releasing defense-related plant hormones (salicylic acid, ethylene, and jasmonic acid) that restrict microbes’ replication, growth, and activities. Some physiological and structural defenses are induced in the infected cells, like thickening cell walls, senescence induction, programmed cell death, and callose deposition [42]. Both necrotrophic and biotrophic microorganisms (pathogenic and non-pathogenic) induce plant immunity. Among the different plant hormones, salicylic acid is the predominant chemical specialized with host response against biotrophic microbes, whereas necrotrophic cause ethylene and jasmonic defense plant hormones [43].

## 4. Molecular Mimicking to Interfere with Plant Immune Functions

Fungal proteins act as molecular mimics to intervene in the plant defense system through derangement of vital plant protein interrelations and exhibit a mechanism of escaping defense induced by MAMP. It has been revealed that MAMP-suppressed genes in fungal colonizing biocontrol have a potent auxin signature [44] that can be beneficent for impeccably equalizing defense-triggering and growth-enhancing activities of symbiotic microbes in the rhizosphere through double role for auxin signaling. WSC3 genes and FGB1 obtained from rhizosphere endophyte *Piriformospora indica* code for a type of MAMP, β-glucan-binding lectin that effectively represses β-glucan induced immunity in numerous plants through modifying composition and properties of the cell wall of fungi, thereby evading the defense signals indirectly [45].

Besides the mechanism mentioned above, few ISR-triggering and growth-enhancing fungi have secured high levels of tolerance against antimicrobial compounds like coumarin scopoletin secreted in reaction to host plant immunity that results in selective inhibition of rhizosphere fungi without impacting the fungal biocontrol. Furthermore, the roots of plants can cause damage to cells to generate a local and robust defense reaction resulting from the increased number of beneficial microbes besides damage-causing pathogenic bacteria [46]. In the case of increased colonization caused by beneficent endophytes in the roots of seedlings, very few MAMP reactions were found in differentiated undamaged roots. Nonetheless, ablation of the cells and root colonization neighboring cells in plants showed MAMP reaction to endophytes. Therefore, though MTI surveillance evading endophytes can trigger MAMP signals from the immune system of plant cells, no cell damage was observed initially by root colonizing pathogens, nor was any robust MAMP response revealed. Nevertheless, the progression of infection ultimately led to the death of some epidermal cells, resulting in the upregulation of MAMP reaction locally in neighboring plant cells. Defense responses to non-pathogenic colonizers of roots having tolerant responses are minimized by damage gating of roots in plants. In the case of pathogens having intrinsic damaging impacts, host plants can instantly recognize the destroyed cells. They can effectively trigger the “switch” of the defense system with “no tolerance behavior” to hamper the progression of infection caused by localized pathogens [46].

Chitin, present in the cell wall of fungi, is a polymeric substance having nonbranched β 1, 4 N-acetylglucosamine molecules that release Chito-oligosaccharides when broken down. Plant receptors identify both the chitin and its products and induce host defense. Chitin is reported as a microbe-associated molecular pattern that triggers host immunity [47], termed pattern-triggered immunity. It has been reported that fungal mycelia produce deacetylase enzymes that promote chitin deacetylation when fungi are present outside the plant; however, deacetylase enzyme-producing genes are repressed if the fungi enter the host. Therefore, fungi mainly express acetylated chitin endogenously in host tissues to ensure their existence. Chitin is converted into chitosan during deacetylation; hence, the host plant does not induce PTI [48].

## 5. Secretion of Apoplastic Proteins and Nucleotides to Promote Colonization

The rhizosphere fungi generate apoplastic communication, which is vital for modulating the microbial settlement by defending the plant’s defense signals. The release of small effector-like proteins helps the endophytes change the host plant’s physiological condition and supports colonization (Table 1) [49]. In addition, lipids, polysaccharides, creecimeinto regulators (auxins, cytokinins), enzymes, and fatty acids, among other factors, work together to ensure that the biocontrol fungi not only penetrate the roots of plants but also maintain and grow effectively to promote plant growth, defend against pathogens and activate active plant resistance mechanisms. These apoplastic proteins are vital in stress response, signaling and immunity, primary and secondary metabolism, and host cell wall structure [50]. Plant’s first line of MTI defense is eluded by the release of effector proteins by colonizing microbes through a regular protein secretion system termed the endoplasmic Golgi reticulum pathway in the apoplast region where interaction occurs with the molecular targets, or these are transported into the cytoplasm for blocking downstream signals. It has been revealed by applying proteomic tools that apoplastic proteins released by fungal biocontrol agents have the potential to suppress the host immune system through complicated physiological processes during the interaction of maize with *Trichoderma virens* [50].

Furthermore, *T. virens* released proteins are primarily related to hydrolysis of cell walls, scavenging of secondary metabolism, and ROS besides presumed effector type proteins. Additionally, proteins like ethylene protein synthetic precursor, cell wall alteration including GHs (glycosyl hydrolases proteins), and antioxidant released proteins, nutrient uptake, and signal transmission involved in the plant hormone signaling have been identified. Plants determine the drastic differences in the expression levels of released proteins [49]. Furthermore, exogenous bioactive nucleotides like eATP (extracellular ATP) that trigger Ca^2+^ response, expression of immunity genes, MAPK activation, and its insight DORN1 both have leading roles in resisting biotic stress and improving plant growth [51]. In addition, an effector protein of the apoplastic fluid, E5′ NT (ecto-5′-nucleotidase), is released during microbe plant colonization instead of pure culture to resist eATP secretion for endophyte growth. An early accumulation of eATP was observed in apoplastic during the root symbiotic colonization of *Serendipita indica*, a beneficent fungus. In addition, E5′ NT produced by fungi altered host-produced apoplastic nucleotide accumulation to promote fungi colonization in plants [52].

**Table 1:** Effector proteins employed by biocontrol fungi and their functions for root colonization.

Effector	Location	Function	Reference
CFEM proteins	Fungal extracellular membrane	Iron integration, appressorium expansion, redox homeostasis, sensing plant surface	[53]
LysM-like effectors	Cell membrane	Chitin recognition during fungal development, masking chitin oligomer Avoid ligand-PRR bonding Self-defense against chitinases	[54]
CP	Cell wall surface	Bind chitin and masking fungal cell wall recognition	[53]
Expansion-like proteins	Outer membrane	Assisting ingress into the host roots Soften the host cell wall with hydrolytic enzymes	[53]
Hydrophobins	Cell wall proteins Surface active proteins	Fungal adherence Altering root architecture	[55]
Lectin	Middle lamella	Alter fungal cell wall compositions and properties	[56]
WSC-proteins	Cell wall proteins	Increase cellular resistance to cell wall perturbation, oxidation, high osmolarity, and metal ions.	[57]

## 6. Transcriptional Reprogramming of the Plant Hormone Defense Signal Genes

**Figure 3:**
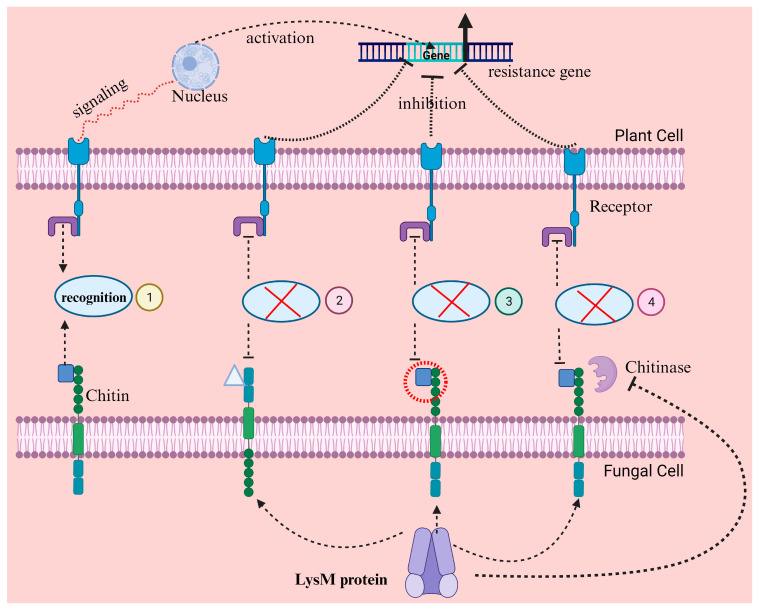
Mechanism of plant immune suppression by fungal LysM effector protein. (1) In the absence of LysM, the chitin polymer of fungal cell wall is recognized by plant cell wall receptors and this recognition results in signaling for the activation resistance genes in plants. LysM effector protein prevents this recognition in three ways. These proteins may alter the chitin composition (2), mask the recognition site of the chitin polymer (3), or secrete chitinase enzymes that degrade chitin polymer (4), resulting in no recognition-by-recognition receptors by plants. Green color circles represent chitin while blue color rectangular represent receptors.

Beneficial microbes associated with plants, such as fungal biocontrols, have developed the potential to elude MAMP identification by directly repressing or bypassing the MAMP-induced reactions of plants’ immunity system that leads to the formulation of host-microbe interaction. This mutual interaction can induce the identification of cell-to-cell signal molecules like PRRs, which recognize the cell wall chitin components. Nonetheless, fungal biocontrol colonizing within host plants has developed numerous mechanisms to elude or repress the host-triggered defense system. Release of effectors is one strategy that intervenes with chitin-induced plant immunity. LysM effector recognized from the fungal species *Rhizophagus irregularis* overturns chitin-induced defense in symbiotic association (Figure 3) [47,54]. LysM effectors in *Trichoderma* fungi play a critical role in evading plant immune recognition [58,59,60]. For example, the Tal6 protein from *Trichoderma atroviride* binds to chitin fragments in the fungal cell wall and sequesters N-acetylglucosamine (GlcNAc) [61]. These chitin fragments, which are microbe-associated molecular patterns, would otherwise be detected by plant pattern recognition receptors, triggering immune responses like the production of chitinases and phytoalexins. By masking these fragments, Tal6 prevents the activation of MAMP-triggered immunity, protecting *Trichoderma* hyphae from plant hydrolytic enzymes and allowing the fungus to establish beneficial interactions with plants, such as mycoparasitism and root colonization [61,62]. This evasion strategy enhances *Trichoderma’s* fitness as a biocontrol agent, enabling it to associate with plants without eliciting strong defense responses [62].

Furthermore, to avoid initial plant immunity reactions, fungal biocontrol drastically modifies the gene transcription of phyto hormone immunity signals that progressed through transitory downregulation of plant defense responses, primarily to permit root colonization [63]. In Arabidopsis plants incubated for 24 h along with fungi, most of the characterized immunity-associated genes were repressed due to SA and JA modulation. Regional repression of root defense systems is a common attribute of ISR-triggering microbes that may assist in the colonization of roots. Notably, during acclimatization to a plant environment, fungal biocontrol releases simultaneous plant-related gene arrays and host plant-shared protein domains through clumping with microbial exogenous mannose and acts as a molecular concealed shroud [63].

## 7. Secretion of the Common Symbiosis Signaling Molecule

Pathogenic microbes and biocontrol agents trigger the second line of defense, the MTI when they invade the plants. Plants cannot differentiate among pathogens or beneficial microbes, and the primary task or interest of the plant is to reduce the inoculum load [64]. This is evident from the considerable overlapping between the immune and symbiotic signal pathways by manipulating cross-regulations among PRR mechanisms of the host. Nevertheless, the plants’ symbiotic and immunity receptors are much alike with few differences; however, they induce completely distinct responses to pathogens and beneficent microbes [65]. Plants mainly employ the cell wall components of microbes to recognize oomycetes and fungal pathogens. Myc factors, the lipopolysaccharide signals triggered by the exudates and cell wall components of microbes, besides small chain oligomers produced by AM fungal pathogens, can be recognized by LysM (lysine motif receptors) that contributed to the induction of CSSP (common symbiotic signal pathways) [66].

In contrast, CSSP, contributed by the host plants for establishing endogenous symbiotic relationships, has yet to be noticed by ECM over the extended evolutionary period [67]. The β-glucans of oomycetes and the component of fungal cell walls in true fungi act as receptors of symbiosis and immunity [65]. The vital signals employed during the microbe-plant interaction are mostly the surface polysaccharides of the microbes, such as lipopolysaccharides, capsular polysaccharides, exopolysaccharides (EPS), and cyclic glucans. In addition, signal molecules from microbes to host plants are vital in reaction to plants. For example, VOCs (volatile organic compounds) that aim at essential points in host physiology trigger the metabolic pathways (downstream) through a domino effect, and in microbe-plant interaction, employed as MAMPs source. Some of these compounds, such as methyl benzoate and m-cresol, are recognized by plant recognition receptors, activating signaling pathways that induce defense responses [67].

## 8. Strategies to Tackle the Rigid Cell Wall of the Host

The primary structure related to the microbe plant communication is the cell walls of host plants. Digestion of the host cell wall, followed by microbe penetration, enables colonization. Identical to the fungal pathogens, fungal biocontrols are also known to secrete CWDE (cell wall degradation enzymes) such as GHs (glycosyl hydrolases) that not only assist in accessing the root tissues but also act as a secreted protein source, which helps the process of communication by releasing signal compounds. In light of the *Trichoderma* genomes, the carbohydrate-active enzyme (CAZymes) that has been characterized belongs to the 136 glycosyl hydrolases families. Any change in the carbohydrate-active enzymes is reflected in the colonization potential of the biocontrol fungi on the host or substrate [68]. The CWDE facilitates the process of colonization and induces the host defense pathways. These CWDEs do not produce any extended destruction to the host cell wall. Instead, they assist in releasing DAMP compounds. Oligo-galacturonide was produced as a DAMP sign as a result of enzymatic processing of endogenous Poly-galacturonases of the fungus *Trichoderma* in association with tomato and Arabidopsis roots and is considered the first recognized DAMP compound [69]. *Trichoderma* produced chitinases and released DAMP compounds in chitooligosaccharides through enzymatic action on polymeric chitin. Although the CWDE has DAMP and MAMP signal compounds, it also enhances the plasticity of root tissues and improves the prolific colonization of roots. In tomato roots, colonization by *T. harzianum* was enhanced by releasing endogenous poly-galacturonase ThPG1. An eight-fold increase in the DNA of the fungus was observed in the root cells employing qPCR, whereas no such increase was seen when ThPG1 expression was silenced in *T. harzianum* [69]. Hence, the molecular interaction between the host roots and the fungal (*Trichoderma*) produced endogenous polygalacturonases that facilitate colonization.

Xylanases, endopolygalacturonases, and cellulases are released by biocontrol fungal endophytes [70]. Seven out of eight genes related to polysaccharide metabolism were over-expressed when tomato or maize plants were cultured with endophytes, improving root colonization [70]. Increasing the expression of genes involved in polysaccharide metabolism would boost the interaction with fungal biocontrol and help the host plant resist environmental and disease stresses. Swollenin and pectinases cause the cell walls to stretch, allowing fungi to grow in areas between the cells by partially breaking down the pectic layer in the middle lamella. As a result, *Trichoderma* increases its activity in the apoplast zone. When CWDEs are secreted by the fungus after contact with the plant’s roots, they help soften the cell wall, but there are too many overlapping functions. Auxins and the quantity of acids in the growing tissue influence how far cells can stretch in the walls. When fungi detect a certain pH employing ion pumps, their signaling pathways support the colonization of plant roots. In contrast, *T. atroviride* decreases the pH, whereas *T. virens* increases it. A higher level of IAA or similar auxin molecules can make roots more flexible and well-developed, helping them penetrate lands. They all contribute to changes in the host’s physical structure [70,71,72,73].

## 9. Host Gene Silencing Through Inter-Kingdom miRNA

**Figure 4:**
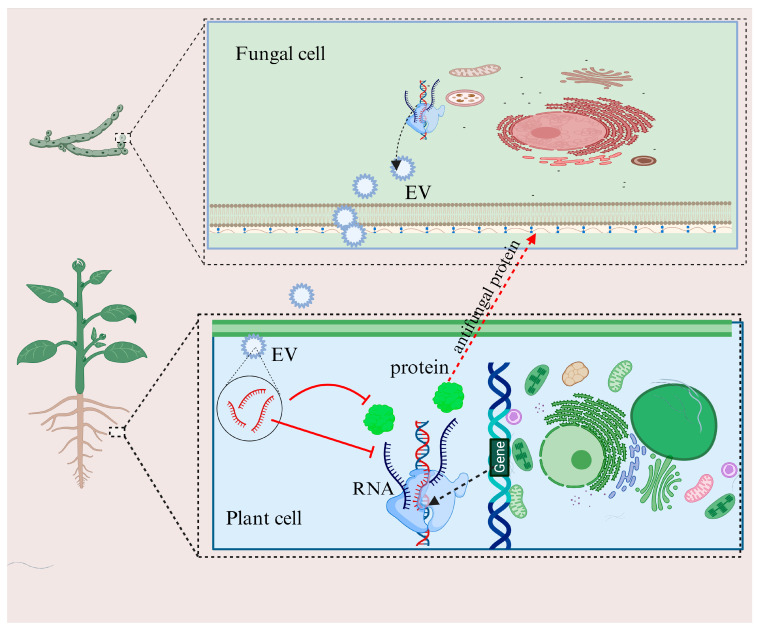
Host gene silencing through inter-kingdom miRNA. The biocontrol fungi export the miRNAs to the host cell through extracellular vesicles (EV). Once inside the host cell, the miRNAs interfere with the host cell RNA and modify the posttranscriptional events, ultimately suppressing the host immunity against fungal colonization.

Small RNAs (sRNAs), despite their short length of 20–30 nucleotides, are essential in playing various biological functions in organisms by dictating gene expression with remarkable specificity. These sRNAs exert control over mRNA by precise base pairing, thus modifying protein biosynthesis through posttranscriptional and translational gene silencing pathways [74]. Exceptionally, they can even play a role in the upregulation of gene expression in certain infrequencies [75]. Among the several sRNA classes, miRNAs are among the most studied. The miRNA synthesis included the initial formation of miRNA precursors that form self-complementary stem-loop structures comprising 70 to 200 nucleotides [76]. These precursors are then changed to mature miRNAs of 21–24 nucleotides by DICER-like (DCL) enzymes [77]. Upon maturation, these miRNAs can interfere with gene expression by targeting transcripts for cleavage, resulting in the downregulation of target genes (Figure 4) [78]. Within the fungal domain, sRNAs, including miRNAs, are recognized for their essential roles in controlling physiological functions in a spectrum of fungi [79]. Ectomycorrhizal fungi, a symbiotic fungus, sometimes play biocontrol action against plant pathogens. They are also assumed to synthesize miRNAs, as they retain the essential components for the sRNA synthesis pathway [80]. sRNAs may be another signaling agent used by biocontrol fungi during symbiosis. Experimental proof of miRNAs regulating plant-fungal interactions has been demonstrated in pathogenic fungi [81]. Recently, a microRNA involved in cross-kingdom gene silencing of the host plant was also reported for the symbiotic fungus *Pisolithus microcarpus* [82].

## 10. Host-Fungal Genetics Contribute to Colonization

Progress has been observed in characterizing many genes that *Trichoderma* requires to grow on and colonize the root system excessively. However, there are many uncertainties, and the colonization procedure needs a better explanation. The genome projects generated genome sequences of *T. reesei* that could destroy cellulose and elucidated the two mycotrophic fungal spp. (*T. virens* and *T. atroviride*) that engage with host plants [83]. These sequences, the primary resources, are beneficial in recognizing the related genes and studying transcriptome and mutant construction. Numerous reverse genetics tools before the *Trichoderma* genome projects have identified some genes that facilitate root colonization. For example, the *T. asperellum* gene, TasSwo translating Swollenin (an expansion-related protein), was characterized by LC-MS from a culture of fungus and seedling of cucumber. Decreased colonization of roots was observed in mutants silenced for Swollenin expressing gene [84]. PG2 strain of *T. virens* is required to trigger ISR; however, it is not a prerequisite for colonization of roots [85]. Surface proteins such as hydrophobins, which are peculiar to fungi, can regulate the penetration or attachment of hyphae of *Trichoderma*. This level is crucial if surface colonization and attachment reduce later root entry capacity. The TasHyd1 gene has been identified as a code for the hydrophobin-related protein employing a discriminative mRNA exhibition assessment of *T. asperellum* hyphae near host plant roots. The surface features were altered when deletion mutant was used, losing their potential to attach and colonize roots. Numerous hydrophobin genes have been demonstrated to play a vital role during the process of colonization of roots [86].

The fungal plant molecular communication can influence the colonization of roots in minimal direct mechanisms. The proteins present on the cell surface or released are vital for colonizing roots by modifying the metabolism or expression of the host gene. Hydrophobins, as mentioned previously, have the potential to change the surface attributes and may behave as signals to the host plant. Hence, a released protein may impact the physical features of the apoplast or plant root surface and act as MAMP to induce or guide the defense system of the host plant. Pathogen or symbiotic agent-secreted proteins change the plant cell functions and are termed effectors, some of which are transported into the host plant cells. The prototype of effectors transported into the host plant cells was initially recognized for oomycetes and bacteria and is currently believed to be highly significant in fungal pathogens; presumably, symbionts utilize identical mechanisms. SSPs (Small secreted proteins) such as effectors of mycorrhiza depict a single class of contenders; tons of SSPs were anticipated from preliminary screening of the three characterized (Sequenced) *Trichoderma* spp. genomes [87]. *Trichoderma* is not a plant pathogen; the fungal effectors that trigger the ISR or other systemic resistances may be the virulent factors, which emerged secondarily to induce resistance without activating the complete plant immunity response. The decline of Sm1 (an SSP) does not result in evidently any defective root colonization. Triggering of ISR and root colonization are different mechanisms. Two other thoughts led us to assume that some overlapping exists between the factors needed for root colonization and effectors that induce ISR. The first consideration is that early access to the fungal biocontrol reveals the host plant to effector molecules that trigger the defense system. Secondly, effectors subdue the defense system to permit the proliferation of the colonization process. The evidence of mycorrhizae strengthens the hypothesis, although the recognition of the factors might be different. Analyses of the three paralogs in *T. virens* to SM1 disclosed that the mutant deficient in one (Sm2) lacked the potential of root colonization in seedlings of corn [88].

Hence, undeniable proofs were revealed for numerous groups of genes in multiple *Trichoderma* spp. that translate to proteins playing a vital role in colonization. The genotype of plants that determines the compatibility or incompatibility of the interaction with fungi is explored widely; however, only a few involve the interaction of plants and *Trichoderma*. Limiting proliferous growth is primarily done by reinforcement of the cell wall. As described above, the Arabidopsis mutant lacking the isochorismate synthase 1 revealed that *Trichoderma* penetrates up to the vascular bundles of the mutant. Considering the host plant side, the primary reason might be the lack of mutant potential to react to the fungi through cell wall reinforcement. Plants lacking the SA-triggered defense mechanism create a more conducive environment where *Trichoderma* reaches the vascular bundles. In the interaction of tomato and *T. harzianum*, it has been shown that colonization of *Trichoderma* is hampered using the SA defense system. In contrast, it was supposedly enhanced in the case of the JA pathway through antagonizing defenses depending on SA [89].

## 11. Factors Affecting Root Colonization

The definition of “root colonization” impeccably pertains to the soil, the natural zone of microbe-plant interaction. During assays related to colonization, host plants are occasionally grown in sand or soil, but often, hydroponic or solidified agar systems are employed using differential synthetic media compositions. The question is whether the different environmental factors impact the biocontrol mechanisms used by the fungal biocontrol for invasion and proficient growth. Is the fungal biocontrol harmful or beneficial to the host plants under the prevalent conditions? In most laboratories, the assay results may not represent the fungus’s behavior towards the host plant in the natural environment. Hence, the results regarding the prevalent micro-environmental conditions and factors impacting fungal and host plant physiology must be analyzed. There was a decrease in the fresh weight of the host *A. thaliana* grown along with *T. asperellum* using sterilized soil; however, a kind impact of *T. asperellum* on *A. thaliana* was recorded when agar media plates were used instead of soil. Furthermore, the study also revealed a strong GUS expression in host primary roots and leaves of DR5: GUS in the Arabidopsis line grown along with *Trichoderma* on PDA media supplemented with tryptophan. On the contrary, when *T. reesei* was grown individually on MS media, it produced a favorable signal in leaves and roots [90].

A split condition was used to investigate the impact of medium composition on the interaction of *Trichoderma* with *A. thaliana* [91]. *T. virens* and *T. atroviride*, without or with immediate contact with host plants, were cultured on PDA or MS medium. The beneficent impact on host plant growth was observed using split systems, primarily on PDA media. *T. atroviride* and *T. virens* succeeded in rescuing *A. thaliana* through root hair production when studied under split conditions on PDA and MS media and upon simple interaction. The severe impact was recorded when *T. virens* was grown on PDA under split conditions, producing more root hairs. The potential of *Trichoderma* to recover the deficiency of root hairs as the result of secretion of VOC (volatile organic compounds) like auxin signal pathway or auxin synthesis precursors. Contrary, a negative impact of retardation after five dpi on primary root elongation was seen following split systems on PDA plates with T. atroviride. This happened due to the increased production of 6-PP in growth mediums related to MS media. Medium composition and strain specificity impacted the production of VOC, which may justify the influence of *Trichoderma* on host plants [91].

## 12. Conclusions

This review has deeply explored the complex molecular tactics utilized by fungal biocontrol agents to successfully establish themselves within plant root systems despite facing the formidable defenses of the host plant. The diverse range of strategies these fungi employ includes altering nucleotide exchange, molecular mimicry, production of apoplastic proteins, and biochemical sensing. These methods enable the fungi to escape the plant’s intrinsic immunity and finely adjust the plant’s defense signaling cascades. Through the modulation of phytohormone pathways and reprogramming of transcriptional responses, biocontrol fungi proficiently change the plant’s immune responses, paving the way for a symbiotic relationship. Furthermore, the review emphasizes the importance of the physical barrier in the form of host cell walls. It elucidates how biocontrol fungi use a combination of mechanical and enzymatic tactics to surmount this obstacle. Additionally, insight into the genetic foundations encompassing host and fungal origins essential in achieving successful root system colonization was discussed. These genetic factors are vital in facilitating the host-fungus interaction, dictating the effectiveness of colonization and the subsequent biocontrol activity. Given the evolving comprehensions from molecular biology, genomics, and ecology, this review article highlights the prospective for a holistic, interdisciplinary approach to improve our understanding of the biocontrol mechanism. Such an assimilated approach is essential for addressing knowledge gaps and harnessing these beneficial fungi in sustainable agriculture. By progressing our grip on the complex interactions between host plants and biocontrol fungi, we can pave the way for advanced strategies to improve crop resilience and production, thus contributing to ecosystem health and food security.
